# Humoral response to HspX and GlcB to previous and recent infection by *Mycobacterium tuberculosis*

**DOI:** 10.1186/1471-2334-7-148

**Published:** 2007-12-31

**Authors:** Marcelo Fouad Rabahi, Ana Paula Junqueira-Kipnis, Michelle Cristina Guerreiro dos Reis, Walter Oelemann, Marcus Barreto Conde

**Affiliations:** 1 Departamento de Clinica Medica, Faculdade de Medicina, Universidade Federal de Goiás, Goiania, Brazil; 2 Departamento de Imunologia, Instituto de Patologia Tropical e Saúde Pública, Laboratório de Imunopatologia das Doenças Infecciosas Universidade Federal de Goiás, Goiania, Brazil; 3 Departamento de Imunologia – Universidade Federal do Rio de Janeiro, Rio de Janeiro, Brazil; 4 Instituto de Doenças do Tórax, Universidade Federal do Rio de Janeiro, Rio de Janeiro, Brazil

## Abstract

**Background:**

Tuberculosis (TB) remains a major world health problem. Around 2 billions of people are infected by *Mycobacterium tuberculosis*, the causal agent of this disease. This fact accounts for a third of the total world population and it is expected that 9 million people will become infected each year. Only approximately 10% of the infected people will develop disease. However, health care workers (HCW) are continually exposed to the bacilli at endemic sites presenting increased chance of becoming sick. The objective of this work was to identify LTBI (latent tuberculosis infection) among all asymptomatic HCW of a Brazilian Central Hospital, in a three year follow up, and evaluate the humoral response among HCW with previous and recent LTBI to recombinant HspX and GlcB from *M. tuberculosis*.

**Methods:**

Four hundred and thirty seven HCW were screened and classified into three different groups according to tuberculin skin test (TST) status: uninfected, previous LTBI and recent LTBI. ELISA test were performed to determine the humoral immune response to HspX and GlcB.

**Results:**

The levels of IgG and IgM against the HspX and GlcB antigens were the same among HCW with recent and previous LTBI, as well as among non infected HCW. However, the IgM levels to HspX was significantly higher among HCW with recent LTBI (OD = 1.52 ± 0.40) than among the uninfected (OD = 1.09 ± 0.50) or subjects with previous LTBI (OD = 0.96 ± 0.51) (p < 0.001).

**Conclusion:**

IgG and IgM humoral responses to GlcB antigens were similar amongst all studied groups; nevertheless IgM levels against HspX were higher among the recent LTBI/HCW.

## Background

Tuberculosis (TB) remains one of the world's major public health problems. The World Health Organization (WHO) estimated 8.9 million of new cases of TB in 2004 and that one third of the world's population is infected by *Mycobacterium tuberculosis (M. tb) *[1,2]. Although the identification and cure of active, infectious cases of pulmonary TB is the most cost-effective public health measure for the control of the disease, the detection and treatment of individuals with latent TB infection (LTBI) may also provide an important role in the fight against the TB epidemic [2]. Different studies have demonstrated that among subjects with recent LTBI, the risk for developing active TB in the first year of follow up was 8 times higher than in the subsequent seven years and that 82% of TB cases developed active disease within 2 years of infection [3,4]. The most common test used to determine if a person has been infected by *M. tb *is the tuberculin skin test (TST). Although of low cost and relatively simple to administer, the TST suffers from a number of well-documented performance and logistical problems, such as the need for individuals to return for test reading, the variability and subjectivity in test application and reading and low specificity [5,6].

In 2005, the FDA approved an IFN-γ assay for the diagnosis of LTBI. In this test, an enzyme-linked immunosorbent assay (ELISA) detects the IFN-γ in fresh blood of sensitized individuals when incubated with a specific antigen [7-9]. A meta-analysis of 75 studies evaluating the IFN-γ assay for LTBI diagnosis suggests that the use of RD1 ESAT-6 and CFP-10 antigens can provide identification of LTBI in individuals with high risk of active disease [8]. Furthermore, less cross-reaction with BCG vaccine and other mycobacteria and the superior test management is in contrast to the variability of administration and interpretation of the TST. Even though the IFN-γ assay presents higher specificity than the TST, this improved specificity is observed only in low-TB-incidence settings. More over, both tests (TST and IFN-γ assay) are not capable of distinguishing recent from previous (presumable old) latent *M. tuberculosis *infection.

Immunological methods are attractive as screening or as diagnostic tests for TB disease or infection because they could be relatively rapid and simple. Challenges for development of an effective immunological test include avoiding cross-reactivity with BCG or mycobacteria other than *M. tb*; consistent performance in genetically and immunologically diverse populations; and the need to discriminate active TB from LTBI, as well as recent LTBI from previous (old) LTBI. Serological ELISA tests can be candidates for the screening of individuals at higher risk to develop active TB, because they are rapid, usually present a higher sensitivity and could be performed at lower income countries due to their low cost. A significant number of *M. tb *proteins induce specific humoral and cellular immune responses and these antigens have been shown to correlate with proven human *M. tb *infection or disease [10-14]. Recent findings suggest that antigens like 16-kDa (HspX, Rv2031c) and 80-kDa antigen (GlcB, Rv1837c) could have a specific response in individuals with high risk of *M. tb *infection with recent LTBI [12-14].

Brazil presents one of the highest TB incidences in Latin America (60/100,000) and is among the 22 countries with the highest TB prevalence. Goiania, central Brazil, a mid Brazilian city with one million people, presented in 2001 an incidence rate of 22/100,000. Although Goiania had a reduced incidence rate when compared to the rest of the country, the State Reference Hospital for Infectious disease (HDT) was the only hospital admitting TB cases from the Central region of Brazil, which received a total of 350 TB patients in 2001, where 77% of them were smear positive [15,16]. Because of that, health care workers (HCW) caring TB patients at HDT could be at higher risk to become infected with TB and consequently with a superior chance to develop active TB.

The purpose of the present study was to evaluate the specific humoral response to HspX and GlcB among HCW with previous and recent latent infection by *M. tb*.

## Methods

### Subject enrollment

The study setting was the TB State Reference center for infectious disease: Hospital de Doenças Tropicais (HDT). At the occasion of the enrollment, June 2001, a cross-sectional study was conducted to determine the prevalence of LTBI among all asymptomatic HCW of HDT age 18 years and older without known history of active TB. After the screening, HCW with no LTBI were prospectively enrolled in a cohort study and were followed through 2002, 2003 and 2004 to determine the incidence LTBI within one year. The complete study was approved by the Ethics Committee of HDT and an informed written consent was obtained from all study participants.

### Clinical evaluation

During the cross-sectional evaluation, the medical history of all HCW was obtained, including information on BCG vaccination (during childhood or re-vaccination) and past TB diagnosis or treatment. A physical examination was performed. All HCW underwent a two-step TST applied to the forearm using the Mantoux technique and read by a trained and certificated professional (degree of intra-reader reliability > 95% and inter-reader reliability ≥ 80%). All testing was performed using purified protein derivative (PPD), RT23 (0.1 ml = 2 TU) (Staten Serum Institute, Copenhagen, Denmark). The first TST was read 48–72 h after application. HCWs with a reading on the first TST of ≤ 9 mm induration were classified as negative and the subject was invited to return for a second TST applied 2 weeks after the first TST. Boosting reaction was defined as having a reaction of ≥ 10 mm on the second TST with an increase in induration of at least 6 mm compared to first TST [3,4]. Presence of an induration of ≥ 10 mm in the first TST or a boosting in the second TST defined the HCW as a case of previous LTBI. Subjects with a negative result in the first TST and no boosting in the second were considered not infected by *M. tb *(uninfected HCWs) and were enrolled in the prospective cohort. All enrolled participants of the cross-sectional evaluation and of the prospective cohort had a sample of venous blood drawn and placed into glass tubes without preservative or anticoagulant for serum separation and into EDTA-coated tubes for whole blood and plasma separation. Fasting venous blood samples (5 ml) were collected from each subject at the time of enrollment, stored at -20°C, and thawed only once at the time of the serology assays. All samples were tested for human immunodeficiency virus infection.

All HCWs enrolled in the prospective cohort were evaluated annually. During the annual evaluation a medical history, physical examination and a new TST were performed. HCW presenting or referring to respiratory symptoms ≥ 2 weeks underwent a chest radiograph and had two sputum samples (spontaneous or induced) collected and stained with Ziehl-Neelsen and cultured on Löwenstein-Jensen media. We considered patients with a positive culture for *M. tb *from a respiratory sample as pulmonary TB case. A 10 mm increase in the size of the induration compared to the TST from the previous year was defined as a conversion. For the purpose of this study a HCW with a TST conversion within one year was classified as a recent LTBI case. HCW with no conversion documented and a TST induration ≤ 10 mm were invited to return for a new evaluation and TST annually for the following 3 years (2002, 2003 and 2004). HCW with recent LBTI underwent isoniazid prophylaxis treatment during six months (300 mg/day). HCW that presented TB symptoms and had a sputum stained positive by the Ziehl Neelsen method were treated according to the Brazilian standard regimen I, which is composed of rifampin (600 mg/day), isoniazid (300 mg/day) and pyrazinamide (400 mg/day) [17].

Excluded from the study during the cross-sectional evaluation were HCWs who had a second TST ≥ 10 mm but who did not meet criteria for booster phenomenon (an increase in induration < 6 mm related to first TST). HCWs enrolled in the cohort who had no conversion documented but TST induration ≥ 10 mm, those with positive HIV test and those who missed the yearly evaluation or did not come for TST reading were also excluded of the study.

### Laboratory evaluation

Antigen preparation: Purified recombinant antigen proteins HspX and GlcB from *M. tuberculosis *were obtained from the Colorado State University TB Research (contract number: NIH, NO1-AI-75320). CSU protocols provide purified recombinant protein, free of other *E. coli *protein and LPS contaminations [18].

Serology testing was performed using an in house ELISA technique. Ninety-six-well microtiter plates (NUNC-immunoplate) were coated with 50 μl of recombinant HspX and or GlcB antigens from *M. tuberculosis*, 2.5 μg/ml in 0.015 M carbonate buffer, pH 9.6 (PA, Vetec). Plates were coated overnight at 4°C. After this incubation period, the plates were blocked with 1% skim milk (Nestle, 100 μl/well) in buffered carbonate-bicarbonate pH 9.6 and incubated for another 2 hours at 37°C. After washing 5 times with PBS 0.05% Tween 20 (PA, Merck), sera samples were diluted to 1/100 in PBS and were added in duplicate to the wells and incubated for 2 hours. Next, after an exhaustive washing was performed, 50 μl ml of conjugate solution (peroxidase-labeled anti-human IgM (1/15000, IgM-HRP, ZYMED Laboratories^R^) or peroxidase-labeled anti-human IgG conjugate (1/5000, IgG-HRP, BIO-RAD^R^), was added into each well. Then the plate was incubated for one hour at 37°C, and after washing, 50 μl/well of substrate solution (citrate-phosphate buffer pH 5.1 (PA, Labsynth), OPD (1 μg/ml, Sigma, USA) and hydrogen peroxide 30 vol (PA, Vetec)) were added. Finally, after 15 min in the dark, 50 μl/well of stop solution (sulfuric acid 4 N (PA, Vetec)) was pipetted into each well and the optical density (OD) was measured at 492 nm in ELISA microplate reader (Thermo Labsystem-Multiskan, Flow Laboratories, McLean, Va). Immunoenzymatic assays were performed blindly by a lab technician, and were developed and standardized in our laboratory for the measurement of antibodies (IgM and IgG) against HspX and GlcB antigens. Each test was performed in duplicate, and the mean absorbance of the wells with no antigen was subtracted from those of the wells with the proteins antigens prior to analysis [19]. In order to exclude cross reactivity due to *E. coli *contamination or to the skim milk, the sera were pre absorbed with *E. coli *lysate (Promega) or with skim milk for one hour at 37°C and then analyzed in our in house ELISA technique. There were no cross reactivity affecting the ELISA results (data not shown).

### Statistical analysis

Student's *t*-test was performed to compare the means of continuous variables and Chi-square (or Fisher test when applicable) was used for dichotomous variables. One-way ANOVA was performed to compare the variance of optical density (OD) from different groups. Differences were considered significant at a *P *< 0.05. The analysis was performed using SPSS Version 11.0.

## Results

**Figure 1:**
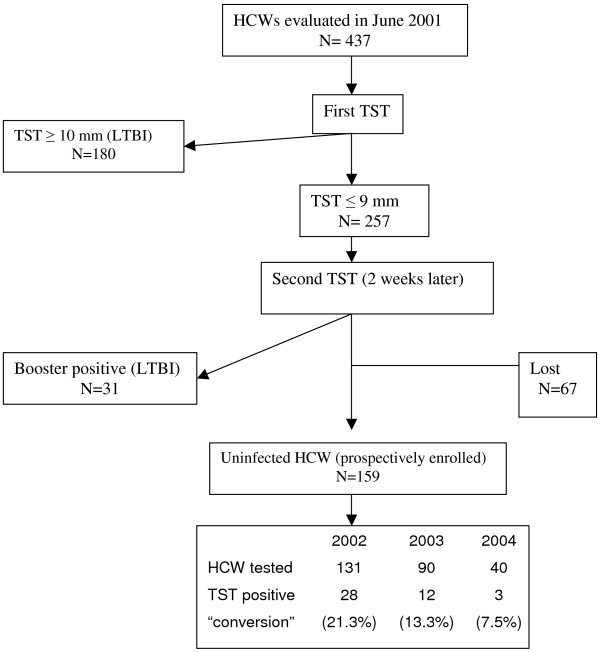
Flow of health care workers evaluated and prospectively enrolled in the study. TST = Tuberculin Skin Test;  LTBI = latent tuberculosis infection;  HCW = health care workers.

Four hundred thirty seven HCW were initially evaluated. A flowchart of screened and followed HCW from June 2001 through June 2004 is presented in Figure 1. The exclusion rate for reasons described above was 13.5% (59/437).

A total of 211 HCW were identified as infected by *M. tb *during the cross-sectional evaluation and were classified as a case of previous LTBI. Sixty seven HCW were excluded because they refused to participate or did not return for the second TST two weeks after the first one. One hundred fifty nine HCW were classified as uninfected and were prospectively enrolled in the study. During the three years of follow up a total of 27% (43/159) of the HCW converted their TST and were classified as a recent LTBI according the study definition. Eighteen percent (28/159) of HCW were lost to follow-up in 2002, 13% (13/103) in 2003 and 49% (38/78) in 2004. The yearly rate of TST conversion was 21.3% (28/131), 13.3% (12/90) and 7.5% (3/40) in 2002, 2003 and 2004, respectively. The demographic data of all HCW is presented in Table 1. Around 80% of all HCW analyzed were female, with an average age of 40.9 ± 10.2 years. Although the BCG vaccination status varied among the groups, the statistical analysis showed that the HCW groups were equally distributed (*P *= 0.505).

**Table 1:** Demographic data and vaccination status of 413 HCWs evaluated at least once during the period of the study.

	Uninfected (n = 159)	Previous LTBI (n = 211)	Recent LTBI (n = 43)
Female/male	137/22	171/40	37/6
Age – range	18–62	18–68	18–62
mean (SD)	39.78 (± 10.3)	41.82 (± 10.2)	39.91 (± 10.8)
			
Previous BCG	100 (62.9%)	163 (77.3%)	30 (59.8%)
old application	92 (92%)	149 (91.4%)	29 (96.6%)
recent application*	8 (8%)	14 (8.6%)	1 (3.4%)

SD = standard deviation; BCG = Bacilli Calmette-Guérin; LTBI = latent tuberculosis infection;

*Vaccination in the last five years

**Figure 2:**
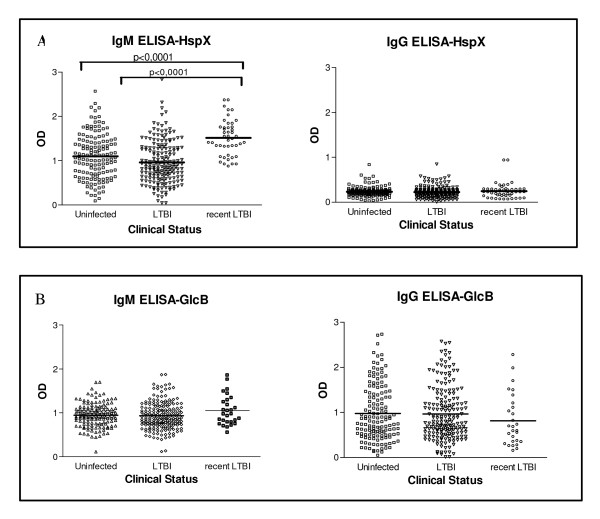
Serologic response of study subjects by indirect ELISA to the recombinant antigens. **A**. Levels of HspX IgM and IgG antibodies in serum from HCW uninfected (n = 159), LTBI (n = 211) and recent LTBI (n = 43). **B**. Levels of GlcB IgM and IgG antibodies in serum from HCW uninfected, LTBI and recent LTBI. Horizontal bars represent the mean antibody levels in the groups. IgM levels in recent LTBI were significantly higher than in those uninfected and LTBI (*P *< 0.0001).

Table 2 presents the response of IgM and IgG to the recombinant antigens in each group evaluated. The humoral response of IgM to HspX was statistically higher among individuals with recent LTBI than among the uninfected (*P *< 0.0001) as well as among individuals with previous LTBI (*P *< 0.0001). There was no difference in the humoral response of IgG to HspX and of IgM and IgG to GlcB among all three groups. Figure 2 shows a dot plot distribution of the humoral response to different antigens among previous LTBI, recent LTBI and uninfected HCW.

**Table 2:** ELISA optical densities with HspX and GlcB recombinant antigens obtained for the different groups investigated.

	HspX		GlcB	
	IgG m ± sd	IgM m ± sd	IgG m ± sd	IgM m ± sd
Uninfected	0.231 ± 0.110	1.090 ± 0.504	0.975 ± 0.614	0.947 ± 0.263
Previous LTBI	0.223 ± 0.121	0.957 ± 0.510	0.977 ± 0.595	0.942 ± 0.314
Recent LTBI	0.252 ± 0.167	1.519 ± 0.394*	0.816 ± 0.574	1.053 ± 0.338

LTBI = latent tuberculosis infection; m = mean of optical density; sd = standard deviation

*IgM against HspX specific humoral responses is significantly higher for recent LTBI than for uninfected (*P *< 0.0001), and LTBI (*P *< 0.0001) individuals. There was no statistical difference between IgG OD against HspX among the different groups. There was no statistical difference between IgG and IgM OD against GlcB.

In order to verify if the BCG vaccination had any effect on the IgM response to these antigens, the response to HspX and GlcB stratified according to their BCG vaccination status among the HCW groups was analyzed. There was no difference between IgM humoral response to HspX (*P *= 0.462) or GlcB (*P *= 0.607) (Table 3 and data not shown). The same inquiry was done regarding IgG responses and no difference was observed (data not shown). HCW that recently converted their TST (rLTBI) also presented IgM levels that did not suffer BCG statuses influence (*P *= 0,567). In addition, the time after BCG vaccination among HCW did not interfere with the humoral responses (*P *= 0,608).

**Table 3:** Optical density of IgM ELISA against HspX obtained for the different groups according to their vaccination status.

	IgM HspX		
	BCG (-) m ± sd	old BCG m ± sd	recent BCG^b ^m ± sd
Uninfected	1.154 ± 0.481	1.108 ± 0.511	0.927 ± 0.535
			
LTBI	1.035 ± 0.622	0.986 ± 0.445	0.991 ± 0.744
r LTBI	1,580 ± 0.505	1,328 ± 0.477	1,560^a^

LTBI = latent tuberculosis infection; m = mean of optical density; sd = standard deviation

^a ^Only one HCW had recent BCG vaccination (last five years).

^b ^Vaccination in the last five years. There was no influence of BCG vaccination to the IgM responses for HspX.

**Figure 3:**
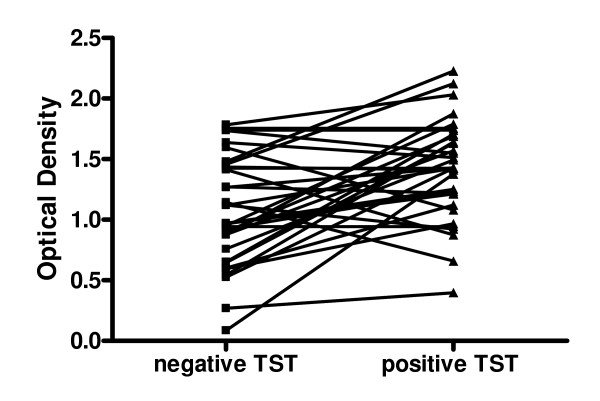
Kinetics of the IgM response against HspX from rLTBI study subjects by indirect ELISA. Negative TST are the OD of the serum samples obtained before the TST conversion and positive TST are the OD at the time of conversion. The lines connects the same person (n = 43). HCW that converted to positive TST presented significantly higher levels of IgM than before conversion (*P *< 0.001).

The sera from rLBTI individuals were analyzed before and after TST conversion. To verify if the TST recent conversion reflected on the IgM response against HspX, a kinetic of the response of the 43 HCW was analyzed and is presented on Figure 3. As expected the IgM levels against HspX increased after TST conversion (*P *< 0.001).

## Discussion

Our study was done in Goiania, a city with one of the best Brazilian quality of life, which receives infectious disease patients from center and mid west region at its State Reference Center for infectious disease: Hospital de Doenças Tropicais (HDT). All hospital workers were invited to participate, 437 out of 540 HDT HCW were enrolled. Although, Goiania's TB incidence was 22/100,000 persons, a low rate for a Brazilian city, an excessively high positive TST rates were observed among the HCW. Two hundred and eleven HCW (48%) were TST positive at the enrollment time and were classified as presenting latent TB infection. This fact could be justified because HDT admitted a median range of ~350 TB cases/year during the five years before the experiment setting, where most of them were smear positive, and also for the reason that the hospital rooms did not present HEPA filters and the HCW did not use protective garments until the time of the study. Similar results were obtained in other Brazilian Hospital [20]. After the cross-sectional study, the HCW were instructed to follow standard operation procedures to avoid the contamination which culminated with a lower TST conversion rates on the yearly follow up. Also in 2003, HEPA filters were installed at the hospital rooms in order to receive TB patients [15,16].

Our findings show that the OD range of IgG and IgM against the HspX and GlcB antigens was the same among HCW with recent and previous LTBI, as well as among non infected HCW (Table 2). Also, Figure 2A clearly shows that the O.D. values of HCW with recent LTBI were in the range detected among subjects with previous LTBI and even in the uninfected group. On the basis of these findings the definition of a cut-off value discriminating recent TBLI from previous or uninfected HCW would not be feasible. This result is similar to the findings of Davidow et al (2005) that evaluated the IgG humoral response to six *M. tb *antigens (including HspX) in 53 individuals with active TB, 218 with inactive TB, 32 with LTBI and 50 uninfected subjects (asymptomatic subjects with negative TST). They showed that the uninfected subjects and individuals with LTBI were serologically indistinguishable [14]. Also, a study using sera in a setting with a high prevalence of TB showed the same conclusion in regards to the humoral response of IgG to the same antigen (HspX or α-crystallin) among active TB patients and TB contacts without TST results [10].

The mean IgM OD level for HspX among HCW with recent LTBI (OD = 1,519) was significantly higher (p < 0,001) than among HCW previously infected (OD = 0.957) and not infected (OD = 1.090), suggesting a possible role of this antigen in identifying recent TB infection. To emphasize this hypothesis, the kinetic of the response by recent LTBI individuals showed an increase in IgM levels against HspX as a consequence of the TST conversion (Fig. 3). A study evaluating the immune cellular response to the antigen HspX reported an association between the interferon gamma production to antigen HspX and TB latency [13]. Another study demonstrated that the cellular immune response and the humoral IgM production start earlier than the ability of the host to produce IgG against this antigen. Thus, different stages of TB may be characterized by a particular antibody or cellular immune response profile against several antigens. Conde et al (2004) reported different responses using IgA and IgG to Mycobacterial P-90 antigen for the diagnosis of active pulmonary TB. They also showed a relatively high IgA humoral response among healthy close contacts when 54% of active pulmonary TB patients presented positive results compared with a low response among healthy controls when only 8% presented a positive ELISA result [21]. In addition, the clinical state of the disease can affect the specific humoral response. Kasermann et al (2005), analyzing pleural fluid of TB patients with pleurisy, demonstrated the presence of specific IgA against MPT-64 and MT-10.3 in the pleural fluid and the authors suggested that this test can be used for the diagnosis of pleural TB [22].

Even though different humoral responses to *M. tb *antigens at different stages of active TB disease have already been well demonstrated, to our knowledge, this is the first report of a specific humoral response to HspX in recent latent *M. tb *infection. Our findings expand the concept that HspX (the 16 kDa antigen, sometimes called 14 kDa) could distinguish recent LTBI from previous LTBI although the range of the optical density was high in both groups. Prior active TB disease or infection with mycobacteria other than *M. tb *might contribute to a low specificity of serological antibody based tests for TB.

Our findings show no significant humoral response (IgM and IgG) to GlcB among the different stages of *M. tb *infection. Singh et al (2005) and Achkar et al (2006) used indirect ELISA to show the presence of GlcB associated with MPT-51 specific antibodies in ~90% of HIV seropositive individuals who developed active TB disease and an absence of response among individuals with LTBI and uninfected subjects. These authors suggested that these specific antibody detections could serve as markers to identify incipient infection with *M. tb*. [23,24]. However, these reports used a combination ELISA measuring specific IgG and IgA to these antigens. Furthermore, there was no discrimination between TST positive and TST negative individuals; in that case the LTBI subjects were not well classified. In our study, we evaluated isolated IgM and IgG humoral immune response to HspX and GlcB.

It has already been reported that the vaccination with *M. bovis *bacillus Calmette-Guerin (BCG) may contribute to a low specificity of serological tests for TB [25,26]. Mori et al (2004) examined a whole blood IFN-γ test using ESAT6 and CFP10 in 216 healthy BCG-vaccinated Japanese adults and 118 patients with TB [27]. The results demonstrated that this test was highly specific (98%) and sensitive (89%) for *M. tb *infection and was unaffected by BCG vaccination [27]. Although it has been shown elsewhere that HspX accumulates in long-term stationary-phase cultures of *M. bovis- *BCG [28], our findings suggest no interference of BCG vaccination in the IgM humoral response to HspX. Using the knowledge that BCG affects the TST conversion during five years after the vaccination [3,4], the results of the groups studied here were compared based on the time of their BCG vaccination (Table 3). HCW with recent BCG vaccination (less than five years) of uninfected, LTBI and rLTBI individuals did not changed the humoral response to the recombinant antigens HspX and GlcB.

The present study has several limitations. The serologic tests were performed retrospectively, using serum that had been stored. Although no serum had been thawed more than once, we cannot formally exclude the possibility that serum storage conditions adversely affected the test performance, although we believe that this is unlikely. The cross reactivity to *E. coli *proteins or to the skim milk were excluded during the standardization. The definition used in the study for recent LTBI might not be appropriate as it is not possible to distinguish through a yearly TST if the *M. tb *infection occurred 2 weeks or 12 months prior to the test. New studies evaluating the response to HspX among TST conversion documented within 12 weeks after a negative TST (such as during the evaluation of close contacts of an active pulmonary TB case) instead of 12 months could help us to better understand the role of this antigen in recent LTBI.

## Conclusion

We conclude that the OD range of IgG and IgM against antigens HspX and GlcB was the same among subjects with recent and previous LTBI, as well as among non infected individuals. However, the OD mean of IgM response to HspX was significantly different among the different stages of *M. tb *infection. This finding suggests a possible use of HspX as a marker for recent LTBI, even in a BCG vaccinated population, perhaps using different techniques to evaluate the antigen response. New studies are necessary to evaluate this hypothesis.

## Competing interests

The author(s) declare that they have no competing interests.

## Authors' contributions

MFR and MCGR carried out the ELISA studies, participated in the analysis of the results and drafted the manuscript. WO participated in the design of the study and performed the statistical analysis. APJK and MBC conceived of the study, and participated in its design and coordination and helped to draft the manuscript. All authors read and approved the final manuscript.

## Pre-publication history

The pre-publication history for this paper can be accessed here:


